# Overexpression of the aphid-induced serine protease inhibitor *CI2c* gene in barley affects the generalist green peach aphid, not the specialist bird cherry-oat aphid

**DOI:** 10.1371/journal.pone.0193816

**Published:** 2018-03-19

**Authors:** Aleksandra Losvik, Lisa Beste, Jennifer Stephens, Lisbeth Jonsson

**Affiliations:** 1 Department of Ecology, Environment and Plant Sciences, Stockholm University, Stockholm, Sweden; 2 Cell and Molecular Science, James Hutton Institute, Invergowrie, Dundee, United Kingdom; Instituto de Biologia Molecular y Celular de Plantas, SPAIN

## Abstract

Aphids are serious pests in crop plants. In an effort to identify plant genes controlling resistance against aphids, we have here studied a protease inhibitor, CI2c in barley (*Hordeum vulgare* L.). The *CI2c* gene was earlier shown to be upregulated by herbivory of the bird cherry-oat aphid *(Rhopalosiphum padi* L.*)* in barley genotypes with moderate resistance against this aphid, but not in susceptible lines. We hypothesized that CI2c contributes to the resistance. To test this idea, cDNA encoding *CI2c* was overexpressed in barley and bioassays were carried out with *R*. *padi*. For comparison, tests were carried out with the green peach aphid (*Myzus persicae* Sulzer), for which barley is a poor host. The performance of *R*. *padi* was not different on the *CI2c*-overexpressing lines in comparison to controls in test monitoring behavior and fecundity. *M*. *persicae* preference was affected as shown in the choice test, this species moved away from control plants, but remained on the *CI2c*-overexpressing lines. *R*. *padi*-induced responses related to defense were repressed in the overexpressing lines as compared to in control plants or the moderately resistant genotypes. A putative susceptibility gene, coding for a β-1,3-glucanase was more strongly induced by aphids in one of the *CI2c*-overexpressing lines. The results indicate that the CI2c inhibitor in overexpressing lines affects aphid-induced responses by suppressing defense. This is of little consequence to the specialist *R*.*padi*, but causes lower non-host resistance towards the generalist *M*. *persicae* in barley.

## Introduction

Aphids are important crop pests. They develop populations of high densities in a short time, often cause symptoms of chlorosis and necrosis, and act as vectors of plant viruses [1]. In cereals such as wheat and barley, the bird cherry-oat aphid (BCA) (*Rhopalosiphum padi* L.) is a cosmopolitan pest in temperate regions. This aphid causes reduction of plant growth without apparent symptoms [1,2], and acts as a vector of the barley yellow dwarf viruses [3,4]. There is a potential to limit virus transmission by breeding for aphid resistance. However, to our knowledge, and as outlined in [5], breeding did not yet result in any commercial barley cultivar resistant against BCA and management relies heavily on treatments with insecticides.

Breeding has produced a number of doubled haploid breeding lines with moderate resistance against BCA [6,7]. The lines are offspring in several generations from a cross of a wild barley (*Hordeum vulgare* ssp. *spontaneum)* accession with the cultivar Lina and backcrosses either with Lina or another cultivar, Barke [7]. The purpose of the present work was to use this material to identify barley genes that add to moderate aphid resistance and could be useful in molecular breeding. The hypothesis was that genes induced by aphids confer increased aphid resistance, more specifically, that genes induced by BCA feeding in moderately resistant, but not in susceptible barley lines confer increased BCA resistance. The approach is supported from studies with other species of plants and aphids where it has been shown that aphid-induced genes not belonging to the category of classical R genes [8] may function to increase resistance to the green peach aphid (GPA) (*Myzus persicae* Sulzer) [9–11].

The gene selected for study in this work, *CI2c*, was identified in a microarray study, where barley gene expression was compared between plants with and without BCA infestation [12]. The genotypes studied were the parents of the original cross, a BC2 offspring with moderate BCA resistance and an unrelated susceptible cultivar. *CI2c* belonged to a group of four genes, which were upregulated by BCA in the resistant genotypes but not in the susceptible cultivars. *CI2c* encodes for a protease inhibitor and belongs to a family of six chymotrypsin inhibitor 2 (*CI2*) genes which are part of the *Mla* (powdery mildew) resistance locus [13]. Further support for the idea that *CI2c* plays a role in defense against aphids comes from a study where it was shown induced in barley by salicylic acid and jasmonic acid [14], mediators involved in plant-aphid interactions [15]. At that time it was known as *BCI-7* (*BCI* = Barley Chemically Induced) [14]. In addition to the suggestive evidence above that *CI2c* might be involved in BCA resistance, *CI2c* gene function supported this idea. Protease inhibitors (PIs) are small proteins involved in regulating plant physiological processes and defense responses [16–20] and are often induced during pathogenesis and upon attack by insect herbivores [21–25]. It is well documented that PIs can inhibit insect growth and reproduction by disrupting their digestive physiology [21,26–28]. With regard to aphids, PIs might inhibit aphid salivary or gut proteases during probing, feeding establishment and digestion [29–33]. There is also a possibility that PIs regulate function of plant endogenous proteases implicated in activation or execution of plant stress responses [18,34–37].

Thus, based on the hypothesis that *CI2c* might negatively affect BCA in barley, we cloned the gene and expressed it in the susceptible barley cv. Golden Promise using the constitutive maize-derived *Ubi1* promoter [38]. The effects of transformation were evaluated not only towards BCA, but also towards the generalist GPA, which can feed on plants in more than 40 families [39]. In a recent study, we showed that GPA performance was negatively affected on transgenic Arabidopsis expressing barley *CI2c* [40]. GPA can colonize barley but shows low reproduction on this host [41] and it was of interest to find out if increased levels of the *CI2c* gene product would affect the GPA performance in barley. The main aim of the study was to evaluate whether the *CI2c* gene has the potential to add to aphid resistance in barley. We found no evidence to support this hypothesis, but did show that overexpression of *CI2c* altered the expression of other defense-related genes and plant responses to aphid feeding, and thereby preventing GPA escape from the plants.

## Materials and methods

### Aphid rearing

Individuals of BCA, *Rhopalosiphum padi* L. and GPA, *Myzus persicae* Sulzer were collected in the field near Uppsala, Sweden. BCA was reared on oat (*Avena sativa* L., cv. Kerstin) and GPA on kohlrabi (*Brassica oleracea* L., cv. Delikatess weisser) in a growth chamber at 22°C, 50% humidity and 150 μmol photons m^-2^ s^-1^, with a photoperiod of 16 h light/8 h darkness.

### Plant cultivation

The genotypes used in the study are presented in Table 1.

**Table 1:** Barley genotypes used in the study.

Abbreviation	Description
**Hsp5**	*Hordeum vulgare* ssp. *spontaneum* ‘Canada Park’ collected in Israel. Moderately resistant against BCA [6,12].
**DH28:4**	A doubled haploid (DH) line with the full number 5172–28:4. It is derived from the backcross (with cv. Lina) of an F1 DH line selected from the cross of Hsp5 with cv. Lina. Moderately resistant against BCA [6,7,12].
**CI2c 6–3**	*CI2c* overexpressing lines of barley cv. Golden Promise, containing a single insertion of the transgene, selected as homozygous lines at T2 generation.
**CI2c 6–4**	
**Control**	Transgenic azygous line of barley cv. Golden Promise, which lost the *CI2c* transgene due to segregation. Selected as null-transformant at T2 generation.

Seeds of barley were sown in pots (7 x 7 cm) filled with planting soil (Plugg- och Såjord, Weibulls, Sweden) and transferred to a growth chamber with conditions as described above for aphid rearing. For experiments with BCA, plants were 7 days old (fecundity, choice tests, RNA analysis and enzymatic tests) or 12 days old (life span test). For experiments with GPA, plants were 12 days old.

### Plasmid constructs, plant transformation and selection

RNA extraction and synthesis of the first strand of cDNA were carried out as described in [40]. The ORF encoding *CI2c* was amplified using the primers 5’-CACCATGAGCTGCGCCGCC-3’ and 5’-TTGCAAAGCTAGCTAGCCAATGTGG-3’. For transformation, Platinum Taq High Fidelity DNA polymerase (Invitrogen) was used for the PCR reaction at 94°C for 30 s, 30 cycles at 94°C for 15 s, 55°C for 15 s and 68°C for 1 min followed by 68°C for 7 min. The PCR products were cloned into the Gateway^®^ pCR8/GW/TOPO cloning vector (Invitrogen) and introduced by *att* site LR Gateway^®^ recombination, according to the manufacturer’s instructions (Invitrogen). For constitutive expression under control of the maize *Ubi-1* promoter (JX947345), the PCR products were introduced in the destination vector pBract 214 (provided by Dr Mark Smedley, John Innes Centre, http://www.bract.org/bract.html). The binary vector was transformed into *Agrobacterium tumefaciens* strain AGL1 together with helper plasmid pSoup [42]. The vector was used to transform immature embryos of barley cv. Golden Promise at the FUNGEN facility, James Hutton Institute, Dundee, UK [43]. Barley transformants were selected on medium containing hygromycin (50 μg ml^-1^, Sigma-Aldrich), and analyses were performed on T2 or T3 lines homozygous for a single-gene insertion.

### Infestation experiments for RT-qPCR and enzyme activity analyses

Twenty adult apterous BCAs were transferred to a small plastic cage (5 cm long, 2.5 cm in diameter) mounted on the first leaf of a 7 days old plant. The cage was closed at both ends by a sponge with a slit for the leaf (S1 Fig). Control plants had empty cages mounted on the first leaf. After 48 h, aphids were removed and the part of the leaf in the cage was cut out and frozen in liquid nitrogen. Six replicates were collected, each containing tissue from two plants. The tissue was ground in liquid nitrogen using mortar and pestle and stored at -80°C until used for RNA and protein extraction.

### RNA isolation and RT-qPCR

Total RNA was extracted from uninfested and BCA infested 9 days old primary leaves. RNA extraction, reverse transcription, qPCR conditions and calculations of relative transcript abundance were performed as described in [7]. *Hsp70* and *SF427* were used as reference genes. The primer sequences are shown in S1 Table.

### Enzymatic assays

Total plant proteins were extracted and inhibition assays with added chymotrypsin were carried out as described in [40] with the following changes. During protein isolation, protease inhibitors cocktail (cOmplete^™^, Roche) was added and the supernatants were cleaned on PD MiniTrap G 25 columns (GE Healthcare). The fluorogenic substrate was initially diluted in methanol and the reaction was carried out in 0.1 M Tris-HCl buffer (pH 8.0) containing 10 mM CaCl_2_ [40]. The endogenous protease activity was measured under the same conditions but without added chymotrypsin. For each genotype, three biological replicates, each consisting of two plants were analyzed. All reactions were prepared as triplicates. Enzyme activities were calculated from 10 min of linear initial velocity rates.

### Aphid life span and fecundity tests

In life-span experiments, one apterous adult BCA or GPA was enclosed in a small plastic cage mounted on the second leaf of a 12 days old plant. When an adult produced its first offspring, the adult and all but one nymph were removed. The reproduction of this nymph was monitored during its life span, by daily counting and removing of newborn nymphs. The intrinsic rate of population increase (r_m_) was calculated using a formula by Wyatt and White (1977) [44] as 0.738 (ln Nd)/d, where Nd is the number of progeny produced by an aphid in a period equal to the pre-reproductive time and d is the pre-reproductive time in days. The number of replicates was 9 for BCA and 6 for GPA. Plants were placed in a 4 L polycarbonate cage (10 × 10 × 40 cm) with side holes and top covered with a net to allow for air flow and kept in a growth chamber with conditions described above under aphid rearing. In five-day fecundity experiments, 20 apterous adult aphids were added. BCAs were released on a sponge placed around the base part of 7 days old plants (S1 Fig). GPAs were enclosed in a small plastic cage mounted on the second leaf of a 12 days old plant (S1 Fig). We carried out two independent experiments for each aphid species, each with 6 plants per treatment. Plants were kept as described above for life span tests.

### Aphid choice tests

A control and a transgenic plant were growing in opposite corners in the same pot. The pots were placed in a transparent 4 L cage as described above under aphid fecundity. Aphids were counted five days after addition of twenty adult apterous aphids (n = 6). BCAs were released on a filter paper placed between two 7 days old plants (S1 Fig). GPAs behave restlessly on barley and were added within a small cage mounted on the second leaf of each of a control and transgenic plant in the same pot (S1 Fig). After aphid addition, the cages were sealed with a sponge and they were opened after 24 h. In the follow-up GPA experiment, the adults and their offspring on each plant were counted on a different subset of the plants at 2, 3, 4 and 5 days after the start of the experiment. n = 6 or 9 (for day 5).

### Statistical analysis

Normal distribution of data was analysed using Shapiro-Wilk normality test and was confirmed for fecundity tests and enzymatic assays, but not for aphid settling, life span test and transcript abundance. Differences in aphid fecundity were analysed using one-way ANOVA (*p ≤* 0.05). Results from enzymatic assays were analyzed by two-way ANOVA (fixed factors “line” and “treatment” and their interaction) followed by Tukey HSD as post hoc test at *p ≤* 0.05. Analysis of differences in transcript abundance and life span experiment were performed using Kruskal-Wallis test. If the test showed significant differences (*p ≤* 0.05), Conover test with *p*-values adjustment by Benjamin-Hochberg FDR method was performed as post hoc analysis at *p ≤* 0.05. Differences in transcript abundance with or without aphids were analysed with Mann-Whitney test at *p ≤* 0.05. All statistical analyses were performed with StatPlus Pro v5 for Windows from AnalystSoft Inc, MedCalc Statistical Software version 17.1 (MedCalc Software bvba, Ostend, Belgium; https://www.medcalc.org: 2017) or at www.astatsa.com [45]. The presence of a signal peptide in the TPPE and hordolisin amino acid sequence and proteins’ location were analyzed using SignalP 4.1 and TargetP 1.1 [46,47].

## Results

### Phenotypes and confirmation of *CI2c* transformation

**Fig 1:**
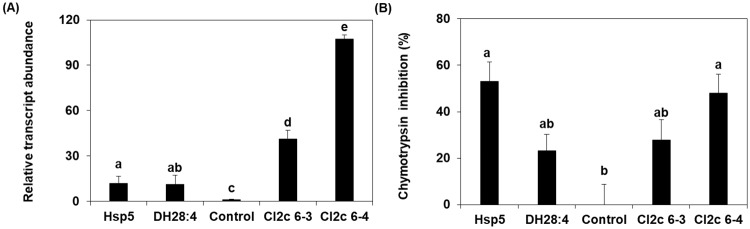
*CI2c* transcript abundance and inhibitory activities in transgenic lines. (A) The relative transcript abundance of the *CI2c* sequence. (B) Chymotrypsin inhibition by plant protein extracts. The results represent the average (±SE) of (A) six biological replicates, three technical replicates, (B) three biological replicates, three technical replicates. Each biological replicate consisted of primary leaf tissue from two plants, 9 days old. The transcript abundance was calculated relative to two reference genes: *Hsp70* and *SF427* and normalized to the control line set as 1.00. Chymotrypsin inhibition was normalized to the control line set as 0% inhibition. Letters indicate significant differences between lines (A: Kruskal-Wallis test, *p*≤0.05; B: one-way ANOVA followed by Tukey HSD post hoc test, *p*≤0.05).

Two transgenic lines were selected for aphid studies, CI2c 6–3 and 6–4. There were no differences in fresh weight or shoot length or any other obvious phenotypic differences between the transgenic lines and control plants (S2 Fig). The *CI2c* transcript abundance was significantly higher in the transgenic lines than in the azygous control or the two moderately resistant genotypes in the background study Hsp5 and DH28:4 (Fig 1A). Line CI2c 6–4 had a higher transcript abundance than line CI2c 6–3 (Fig 1A). The presence of the gene product CI2c was analyzed in enzymatic assays with added chymotrypsin. The inhibitory activity in CI2c 6–4 was significantly higher as compared to in the control line, and the activity in CI2c 6–3 was also higher, although not significantly higher than in the control line. (Fig 1B). The activity in CI2c 6–4 was similar as in Hsp5 and that of CI2c 6–3 similar to in DH28:4.

### Aphid life span and fecundity is not affected on *CI2c* overexpressing lines

The life span and the length of the reproductive life were similar for the two aphid species (Table 2). However, the pre-reproductive period was shorter and the number of nymphs per individual or per reproductive day was much higher for BCA than GPA, as well as the intrinsic rate of reproduction (Table 2). The production of nymphs per day had a broad optimum and declined with time for BCA, whereas for GPA it was low throughout the reproductive life (S3 Fig).

**Table 2:** Life span and reproduction of BCA and GPA on barley plants.

	Pre-reproductive days	Lifespan (days)	Reproductive life (days)	Nymphs/ individual	Nymphs/ reproductive day	r_m_
**BCA**	6.3 ± 0.16	28.9 ± 1.0	12.9 ± 0.6	59.6 ± 3.6	4.6 ± 0.2	0.42 ± 0.01
**GPA**	11.8 ± 0.6	24.4 ± 2.7	12.2 ± 3.2	13.8 ± 3.6	1.2 ± 0.1	0.14 ± 0.04

The results represent mean values (± SE) on the azygous control line. r_m_ = intrinsic rate of population increase. BCA: n = 8; GPA: n = 6.

In fecundity tests starting with adult apterous aphids, the average numbers of BCA on control plants after five days (no cages) were 103 ± 4.9 (SE) and those of GPA (kept in small cages) were 60 ± 5.2 (SE). There were no significant differences in aphid numbers between control and transgenic plants (***p*** > 0.05, one-way ANOVA).

### GPA avoidance is lower on plants overexpressing *CI2c*

**Fig 2:**
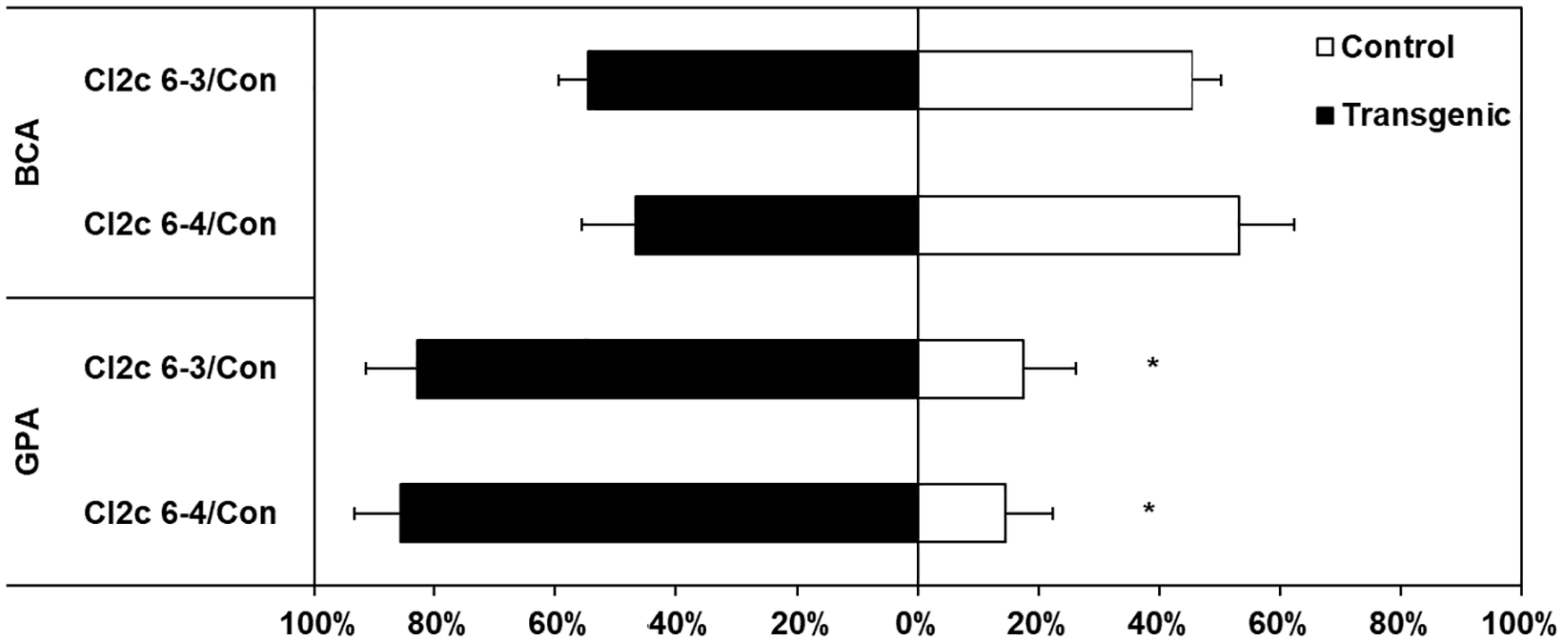
Aphid numbers in choice tests. White bars represent control plants and black bars transgenic plants. Twenty adult apterous BCAs were placed in between a control and a transgenic plant in the same pot. Ten adult apterous GPA were placed within small cages on the control and on the transgenic plant in the same pot and after 24 h, the cages were opened. The total number of aphids on each genotype was counted after 5 days. Results are presented as percentage of total number of aphids counted on each plant pair. Average aphid numbers (±SE) on pair Con/CI2c 6–3 were 136.2 ± 10.2 (BCA) and 7.0 ± 1.9 (GPA) and on Con/CI2c 6–4: 120.7 ± 15.1 (BCA), respectively 22.3 ± 7.0 (GPA). Asterisks indicate significant differences (Wilcoxon matched pair test, *p*≤0.05). n = 6.

**Fig 3:**
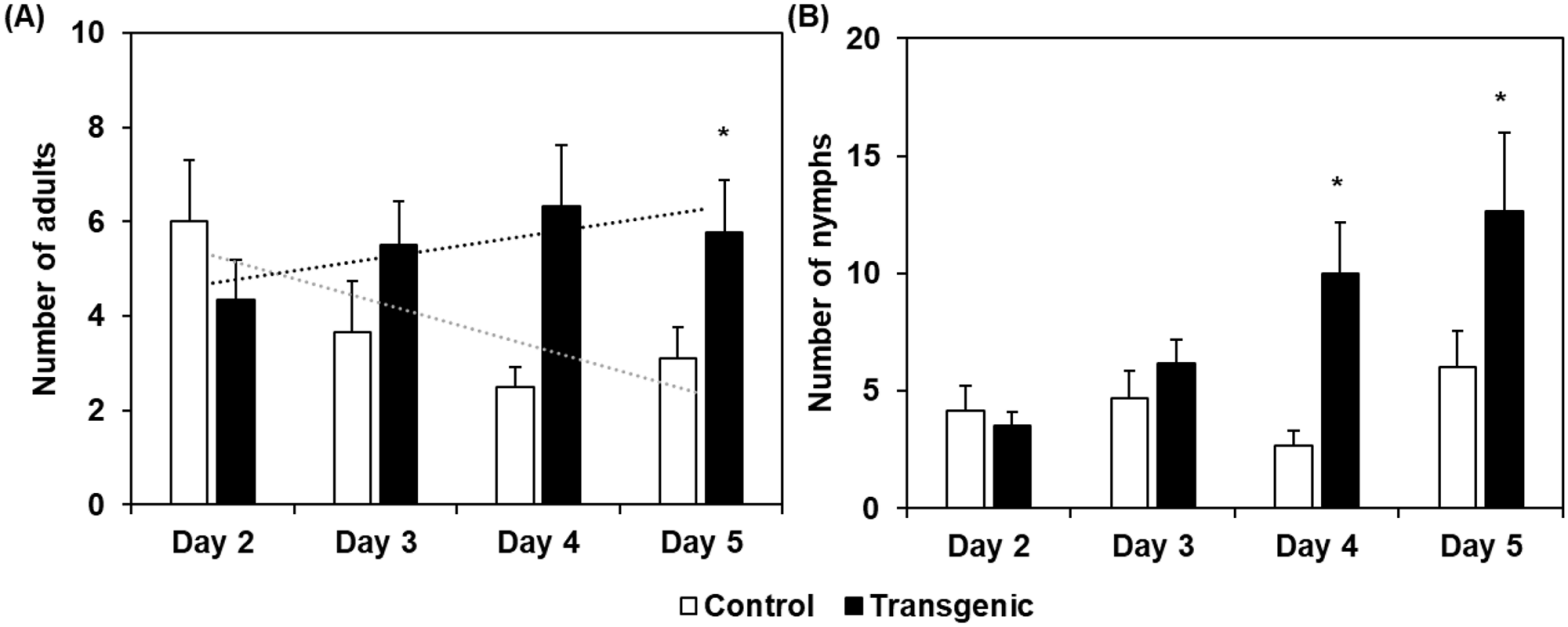
Numbers of GPA adults and nymphs with time in choice tests. White bars represent control plants and black bars transgenic plants. Ten adult apterous GPAs were placed within small cages on control and CI2c 6–4 plants in the same pot. After 24 h, the cages were opened and the aphids were free to move. Adults and nymphs were counted on a subset of the plants each day after the release. Bars show average numbers ± SE. Dotted lines indicate trends. Asterisks indicate significant differences between control and the overexpressing *CI2c* line for a certain day at *p*≤0.05 (Wilcoxon matched pair test). n = 6, except for day 5 where n = 9.

In the five days choice tests, the numbers of BCA were not significantly different on control or *CI2c*-overexpressing plants (Fig 2). In contrast, the numbers of GPA were clearly higher on the lines CI2c 6–3 and CI2c 6–4 as compared to on control plants (Fig 2). We then counted the numbers of GPA adults and nymphs on CI2 6–4 and on control plants each day during four consecutive days. The results showed a trend to lower numbers of adult aphids from day 2 to day 5 on control plants, but a trend for slight increase on CI2c 6–4 (Fig 3A). The number of nymphs remained low on control plants, but increased on the CI2c 6–4 line (Fig 3B).

### Aphid-induced responses are modified in plants overexpressing *CI2c*

**Fig 4:**
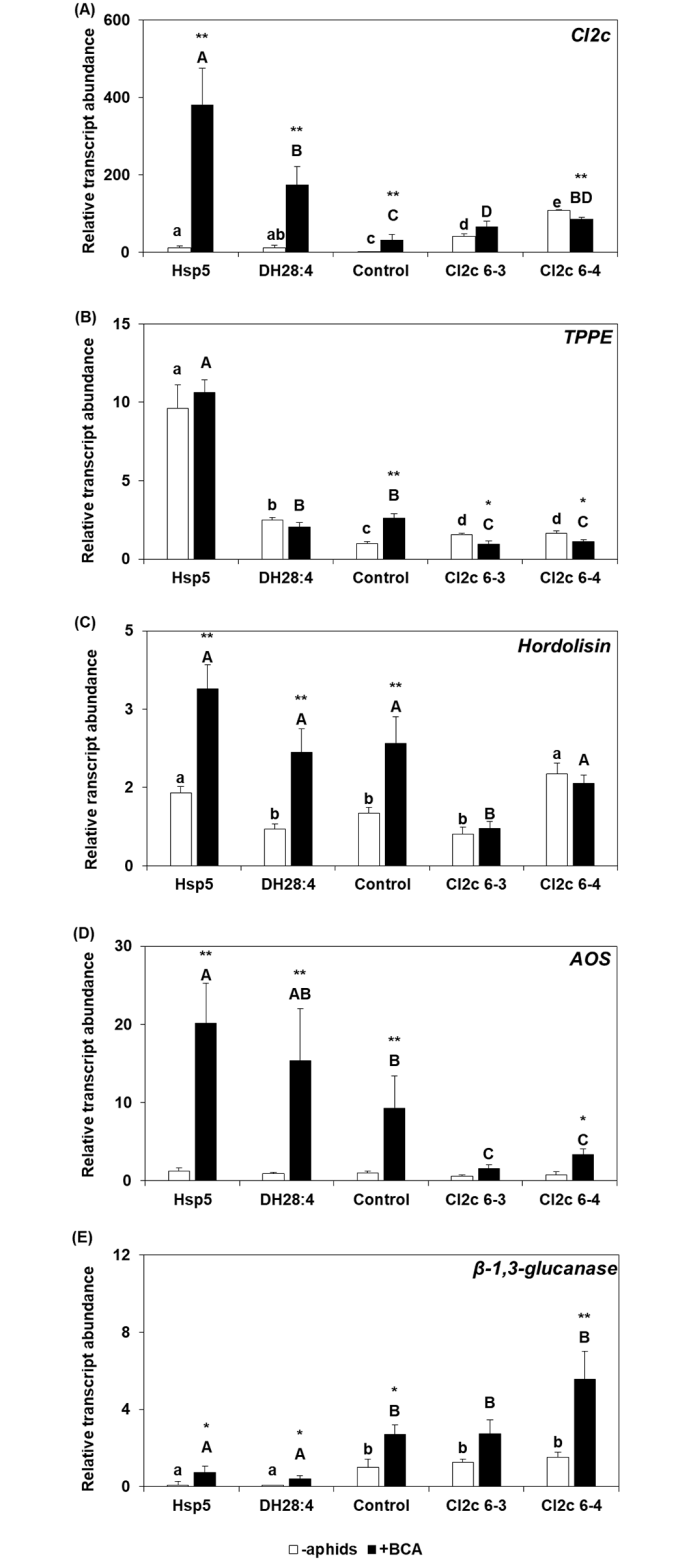
Transcript abundance in barley leaves with and without BCA. Primary leaves were infested during 48 h with twenty adult apterous BCA. White bars represent uninfested plants and black bars aphid-infested plants (±SE). The transcript abundance was calculated relative to the reference genes: *Hsp70* and *SF427* and normalized to uninfested control line set as 1.00. Different letters indicate significant differences between the lines (Kruskal-Wallis test, *p*≤0.05), asterisks indicate significant difference in one genotype with or without aphids (Mann-Whitney test, *p ≤ 0.05, **p ≤ 0.01). Six biological replicates (with two plants each) and three technical replicates.

To investigate aphid-induced responses, five genes were studied: *CI2c*, thionin proprotein-processing enzyme (*TPPE*) [48], a subtilisin-like serine endoprotease, *hordolisin* [49], allene oxide synthase (*AOS*) and a *β*-1,3-glucanase [50]. The genes are induced similarly by BCA and GPA (S4 Fig). All five genes were induced by BCA in the control line (Fig 4), but only *AOS* and *β*-1,3-glucanase were induced in transgenic plants, and only in the line CI2c 6–4. In contrast, *TPPE* was found repressed with BCA in both transgenic lines, and *CI2c* repressed in CI2c 6–4 (Mann-Whitney *p* ≤ 0.05). The results for Hsp5 and DH28:4 showed that *CI2c* was induced by BCA at much higher transcript abundance than in the control line. The pattern of aphid induction was similar as in control for hordolisin and *AOS*. *TPPE* was not induced by BCA in Hsp5 and DH28:4 and the transcript abundance of the *β*-1,3-glucanase was much lower in these genotypes than in control and transgenic lines both before and after aphid infestation.

**Fig 5:**
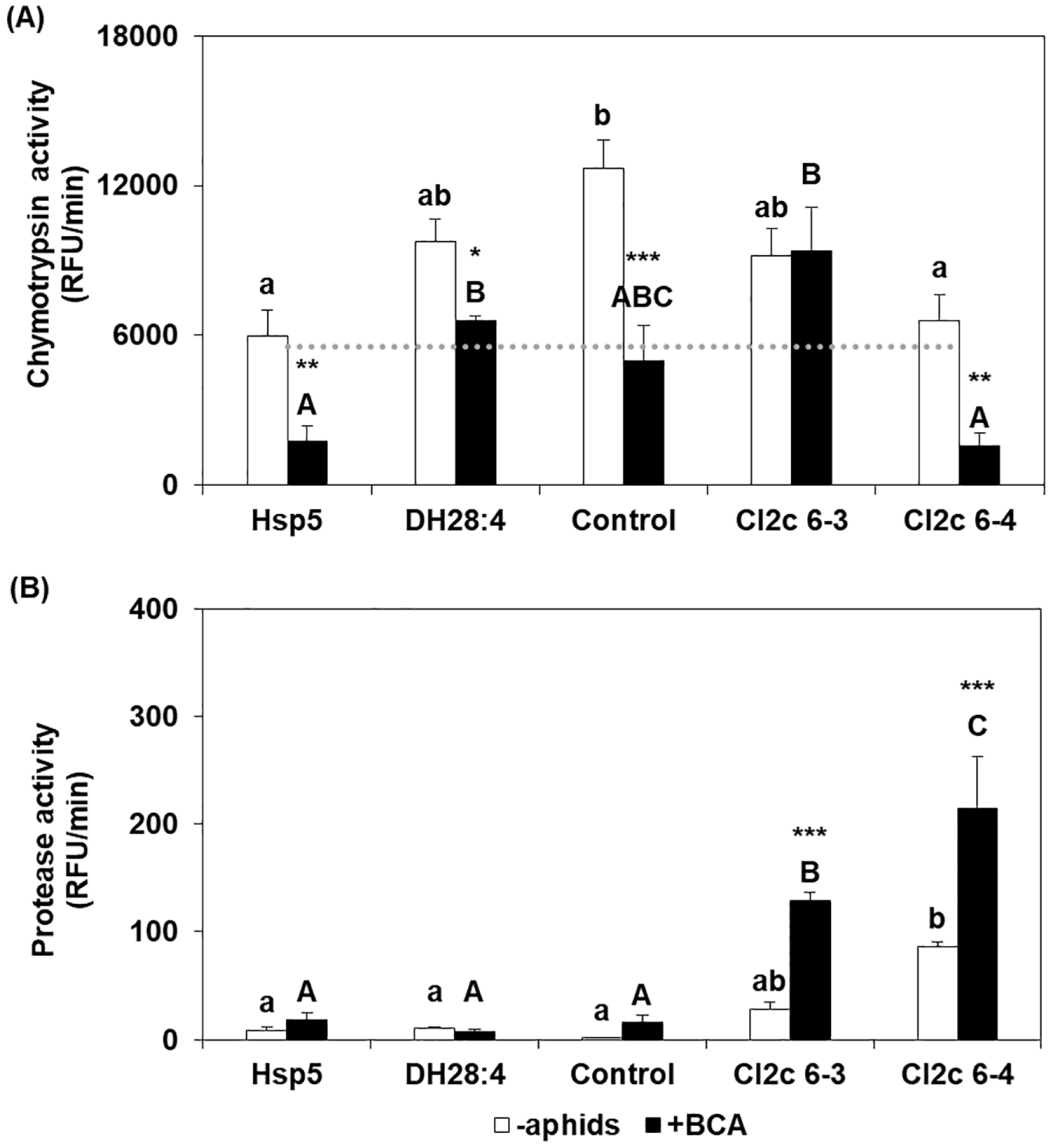
Chymotrypsin inhibition and protease activity in barley leaves with and without BCA. Primary leaves were infested during 48 h with twenty adult apterous BCA. White bars indicate uninfested and black bars infested plants. The results represent the average (±SE) of three biological replicates, each consisting of two plants, and three technical replicates. (A) Chymotrypsin activity. The dotted line indicates chymotrypsin activity in a sample without added plant protein. (B) Activity of endogenous plant proteases. Different letters indicate significant differences between the different genotypes, asterisks indicate significant differences in one genotype with or without aphids (two-way ANOVA with “line” and “treatment” as fixed factors followed by Tukey HSD post hoc test; *p ≤ 0.05, **p ≤ 0.01, ***p ≤ 0.001). RFU = relative fluorescence unit.

Measurements of enzyme activity showed that the chymotrypsin activity was lower (i.e. the inhibitory activity higher) in samples from aphid infested plants as compared to in uninfested plants (*F*_1,20_ = 35.34, *p* < 0.001). The difference was significant for each line, except CI2c 6–3 (Fig 5A). Comparing between the lines, the activity in infested plants was similar in CI2c 6–4 as in Hsp5 and in CI2c 6–3 as in DH28:4, but none of them significantly different from the control line. The endogenous protease activities from aphid-infested plants were significantly higher than in uninfested plants in both the *CI2c* transgenic lines, but not in the other genotypes (Fig 5B). The line CI2c 6–4 showed higher proteolytic activity than non-transformed genotypes also in the uninfested tissue (Fig 5B). It should be noted that the degradation of the fluorogenic substrate was at a much lower velocity with plant endogenous proteases than with added chymotrypsin (Fig 5).

## Discussion

The present study tested the hypothesis that the gene *CI2c* would contribute to BCA moderate resistance in barley, by studying aphid performance in barley lines overexpressing *CI2c*. Transgene expression and translation into a functional protein in the studied lines was confirmed by showing higher constitutive accumulation of *CI2c* transcript as well as higher chymotrypsin inhibitory activity in the transgenic lines than in the azygous control (Fig 1). The results showed that none of the parameters related to BCA life span or fecundity were affected in the *CI2c* overexpressing lines. A second finding was that GPA preferred the overexpressing lines to control lines in tests where these aphids could move between plants (Figs 2 and 3).

The first question is why BCA was not negatively affected on the overexpressing lines. The original resistance bioassays showed ca. 40 and 15% lower increase of aphid nymphal weight in Hsp5 and DH28:4, respectively, as compared to in susceptible cv. Lina [7, 12]. Our present tests were not identical, but since nymphal weight is an indicator of fecundity, any effect should have been revealed by a lower fecundity. The first explanation to consider is that the level of expression of *CI2c* was too low. The constitutive transcript abundance of *CI2c* was much higher in CI2c 6–3 and CI2c 6–4 than in Hsp5 and DH28:4 (Fig 1) and the chymotrypsin inhibitory activity was similar in extracts from the transgenic lines as in extracts from Hsp5 and DH28:4, both in uninfested and aphid infested leaves (Figs 1 and 5A). Therefore, presuming that *CI2c* is responsible for aphid resistance in the overexpressing lines, they would be expected to exhibit similar levels of BCA resistance as Hsp5 and DH28:4. It may however be noted that the level of protease activity in uninfested leaves was higher in the overexpressing lines than in controls (Fig 5B). It is possible that overexpression of *CI2c* leads to induction of as yet unidentified CI2c-insensitive chymotrypsin-like proteases to compensate for the presence of the inhibitor.

Another possible reason for not finding an increase of the BCA resistance is that CI2c acts in coordination with one or more of the three other genes found specifically upregulated by BCA in the moderately resistant lines in the micro-array study. These were encoding, respectively, a lipoxygenase (*LOX2*.*2*), a putative serine/threonine kinase and a calcium-binding EF-hand protein [12]. The fecundity of BCA and GPA was lower in barley overexpressing *LOX2*.*2* [51]. Several jasmonate-regulated genes were more highly expressed in *LOX2*.*2* overexpressors, among them *CI2c* (although not to the same levels as in the *CI2c*-overexpressing lines studied here) [51]. It cannot be excluded that in Hsp5 and DH28:4, the action of the CI2c inhibitor is affected by other gene products induced by a higher *LOX2*.*2* expression. Also, we cannot exclude that the putative serine/threonine kinase or the calcium binding protein affect either CI2c itself or proteases whereupon CI2c is acting. Thus, the present data do not provide the answer whether *CI2c* adds to BCA resistance in Hsp5 or DH28:4. However, they do not give any support to the idea that *CI2c* at higher expression would increase BCA resistance in other barley genotypes, at least not solely.

The second question is why GPA preferred the *CI2c* overexpressing lines when given the choice between them and control plants. This finding was especially unexpected considering that *CI2c* overexpressed in Arabidopsis had a transient inhibitory effect on GPA fecundity [40]. We have suggested that in Arabidopsis, there was a direct effect of the CI2c protein on GPA metabolism or reproduction [40]. In barley different mechanisms seem to be at play. The positive effect of CI2c in barley was only seen when GPA was free to move, not in the tests where it was confined in small cages. Further observations clarified a tendency for GPA to escape from control plants, but not from CI2c 6–4 plants (Fig 3). The avoidance was also illustrated by much higher aphid numbers when GPAs were confined within a cage for five days (ca 60), than when free to move (up to ca 20, Fig 2). The tendency for escape was seen during several days after addition, suggesting that it is not explained by short term deterrence. The phloem composition seems not to be the decisive factor, since GPA fecundity did not differ between control and *CI2c* overexpressing lines in the life span tests, where aphids had established feeding in the phloem. We therefore suggest that the results are best explained by presuming that GPA encounters negative factors during probing and feeding establishment in barley, leading to escape and secondly, that such negative factors are weaker or absent in the *CI2c* overexpressing plants, or balanced by positive factors. As possible negative factors we considered *CI2c*, two proteases (*TPPE* and hordolisin) and *AOS*. *AOS* is involved in the biosynthesis of jasmonic acid and known to be induced by BCA in barley as a general defense response [12]. Their expression was stable or suppressed by aphids in the transgenic lines, when compared to control or resistant plants (Fig 4). As a putative positive factor, we considered a *β*-1,3-glucanase, suggested as a susceptibility factor towards BCA [50]. In our study, it was induced by aphids at the highest transcript levels in the CI2c 6–4 transgenic line (Fig 4). Thus, suggested defense genes were expressed at lower levels and one putative susceptibility gene was expressed at higher levels in the *CI2c* overexpressing plants upon aphid infestation. This indicates that either CI2c itself or proteases whereupon it is acting [52], are affecting general defense responses. Several previous reports show similar effects. As an example, a pathogen-induced P69 subtilisin-like protease from tomato was one of the first to be reported as having a role in plant defense, possibly by activating signal transduction pathways [53]. Further studies showed that P69 homolog, an extracellular SBT3.3 subtilase from *A*. *thaliana* was in fact involved in signaling during immune priming and was required for the expression of genes responding to salicylic acid [54]. In accordance with the above, it was shown that overexpression of the protease inhibitor *BoCPI-1* in broccoli resulted in down-regulation of the expression of a cysteine protease [55]. In contrary to our findings, it was shown that induction of defense genes was compromised in PI-silenced pepper leaves due to down-regulation of genes normally induced during pathogenesis [20]. Although the observed effect was the opposite, it indicates the involvement of protease-PI interaction in fine-tuning of plant responses.

It is also possible that there are direct effects or CI2c or proteases on GPA metabolism or reproduction. The aphids are likely to be exposed to proteases and PIs during tissue penetration, which is mainly extracellular. Analyses of the TPPE and hordolisin amino acid sequences with SignalP 4.1 and TargetP 1.1 [46,47] showed that both proteins have a N-terminal signal peptide of respectively 24 and 25 amino acids directing them to the secretory pathway. The CI2c protein is also believed to be localized extracellularly [40]. Extracellular proteases consumed by GPA during probing might have negative effects on their metabolism. The higher levels of CI2c in the *CI2c-*overexpressing lines, in combination with the suppression of protease induction, would counteract such effects.

The differences in aphid-induced responses between control and transgenic plants would however not be the only reason for the avoidance of GPA on barley, because even on the *CI2c* overexpressing plants, not more than half of the added aphids were recovered. Thus, we presume that other constitutive and/or induced factors than those studied here, are involved in barley-GPA interaction. We found that genes induced in barley by BCA were also induced by GPA (S4 Fig). This is in accordance with a role of the obligatory symbiont *Buchnera aphidicola* as instrumental in triggering plant defenses [56]. The first effector to be clearly identified from aphids, was in the saliva of the potato aphid (*Macrosiphum euphorbiae* Thomas) and this protein was shown to originate from *Buchnera* [56]. However, also the rearing plant is of importance to aphid performance on non-host plants. Earlier studies showed that GPA reared on potato did not survive to reproduce when added on barley, whereas GPA reared on barley survived, but performed poorly [41]. Here, GPA offspring from adults reared on kohlrabi survived with a total life span as long as that of BCA. The number of GPA offspring in a five-day test with aphids confined in a cage, was in the same order of magnitude as that of BCA. The major obstacle for GPA performance on barley appeared to be during probing and feeding establishment, causing escape when the aphids were free to move. An interesting question is what constitutes the mechanism for the different performance depending on the rearing plant. Further studies addressing this issue may reveal new aspects of non-host resistance.

## Conclusions

The overexpression of the serine protease inhibitor *CI2c* in barley has no measurable effect on the performance of the specialist BCA, but prevents escape of the generalist GPA from the plants. The higher levels of CI2c in the transgenic plants modify aphid-induced responses, generally to be suppressed, but for a *β*-1,3-glucanase to be increased. The mechanisms for the effects on aphid-induced responses remain to be investigated, but it is suggested that proteases originating either from aphids or plants are targeted by the inhibitor. The results suggest that induced responses contribute to the escape behavior of GPA on barley.

## Supporting information

S1 Table Primer sequences used in RT-qPCR. (DOCX)

S1 Fig Aphid tests. (DOCX)

S2 Fig Fresh weight and length of barley shoots. (DOCX)

S3 Fig Nymph production per aphid during the lifespan of BCA and GPA. (DOCX)

S4 Fig Transcript abundance of aphid-induced genes in control barley leaves before and after infestation with BCA and GPA. (DOCX)
