# Supplementary material for: Overexpression of the aphid-induced serine protease inhibitor CI2c gene in barley affects the generalist green peach aphid, not the specialist bird cherry-oat aphid
Source: PLoS One. 2018 Mar 19;13(3):e0193816. doi: 10.1371/journal.pone.0193816 (PMC5858787; doi:10.1371/journal.pone.0193816)
Supplement: S1 Table — (DOCX) [file pone.0193816.s001.docx]

| Function | Gene abbreviation/Barley 1 contig | Accession number | Primer sequence |
| --- | --- | --- | --- |
| Proteinase inhibitor | *CI2c* | AJ250663.2 | F AGGTAGCGGGAAAGTCCATC  R GGTCCTGAAGTCGAGGGTCA |
| Thionin  proprotein-processing enzyme | *TPPE* | AK362004.1 | F TGAAGAAGGGCATCGTCGTC  R GCAGCGACAGTCAGTATCCA |
| Hordolisin | *HvHordolisin* | AK368290.1 | F TGCCGTCTCATTTTCCTGCT  R CACACGTTGCCACAACTCAC |
| *β*-1,3-glucanase GII | Contig1637_s_at | AF515785.1 | F TCGCCATGTTCAACGACAACC  R TGCTTGGTTGCACTCTTCC |
| Allene oxide synthase | *AOS*/Contig3097_at | AJ250864.1 | F TACGACACCTTCACGGCCAAAGTC  R ATTTAAACAGCGTCTGCCACACCG |
| Tubulin | *HvTubulin* | U40042.1 | F AGCATGAAGTGGATCCTTGG  R AGTGTCCTGTCCACCCACTC |
| 20S proteasome alpha subunit E | *SF427* | EY965287.1 | F ATTCCAGCCAGCGCGTAGGTAT  R CCATCACCAAGTCGCCTTTAGTAGT |

**S1 Table. Primer sequences used in RT-qPCR**
