# Supplementary material for: Overexpression of the aphid-induced serine protease inhibitor CI2c gene in barley affects the generalist green peach aphid, not the specialist bird cherry-oat aphid
Source: PLoS One. 2018 Mar 19;13(3):e0193816. doi: 10.1371/journal.pone.0193816 (PMC5858787; doi:10.1371/journal.pone.0193816)
Supplement: S1 Fig — (DOCX) [file pone.0193816.s002.docx]

**
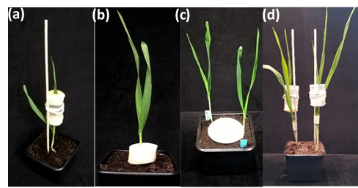
**

**S1 Fig**. **Aphid tests**. All were carried out with the plants in large cages (10 x 10 x 40 cm) not shown in the photos. (a) Life span tests and five day fecundity test with GPA; 48h infestation with BCA (primary leaf); (b) Five day fecundity test with BCA; (c) Choice test with BCA. Aphids were placed in between the two plants; (d) Start of the choice test with GPA. Aphids were placed within the cages and they were opened after 24 h.
