# Supplementary material for: Overexpression of the aphid-induced serine protease inhibitor CI2c gene in barley affects the generalist green peach aphid, not the specialist bird cherry-oat aphid
Source: PLoS One. 2018 Mar 19;13(3):e0193816. doi: 10.1371/journal.pone.0193816 (PMC5858787; doi:10.1371/journal.pone.0193816)
Supplement: S2 Fig — (DOCX) [file pone.0193816.s003.docx]

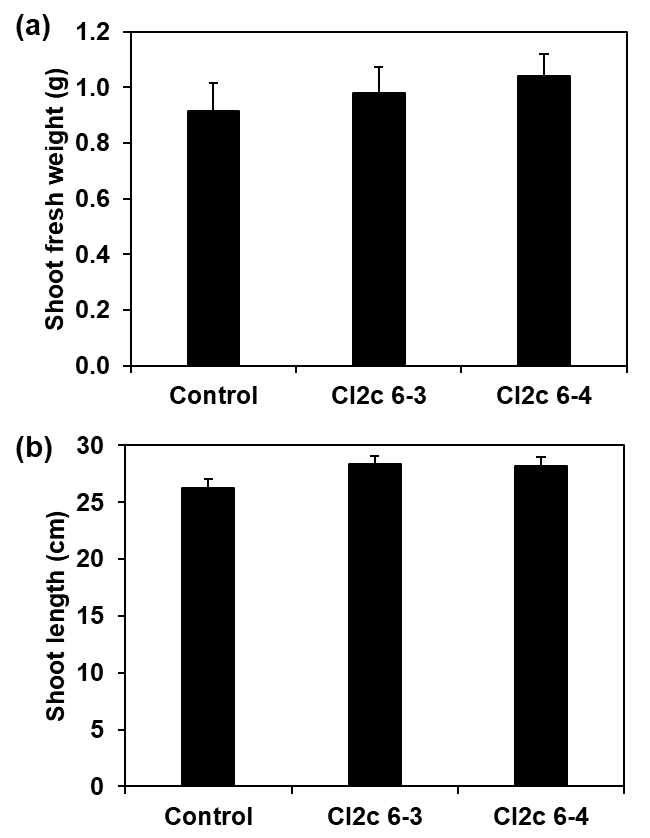
**S2 Fig.** **Fresh weight and length of barley shoots**. (a) Weight, (b) length. Error bars indicate SE; n=12. Plants were 17 days old.
