# Supplementary material for: Overexpression of the aphid-induced serine protease inhibitor CI2c gene in barley affects the generalist green peach aphid, not the specialist bird cherry-oat aphid
Source: PLoS One. 2018 Mar 19;13(3):e0193816. doi: 10.1371/journal.pone.0193816 (PMC5858787; doi:10.1371/journal.pone.0193816)
Supplement: S3 Fig — (DOCX) [file pone.0193816.s004.docx]

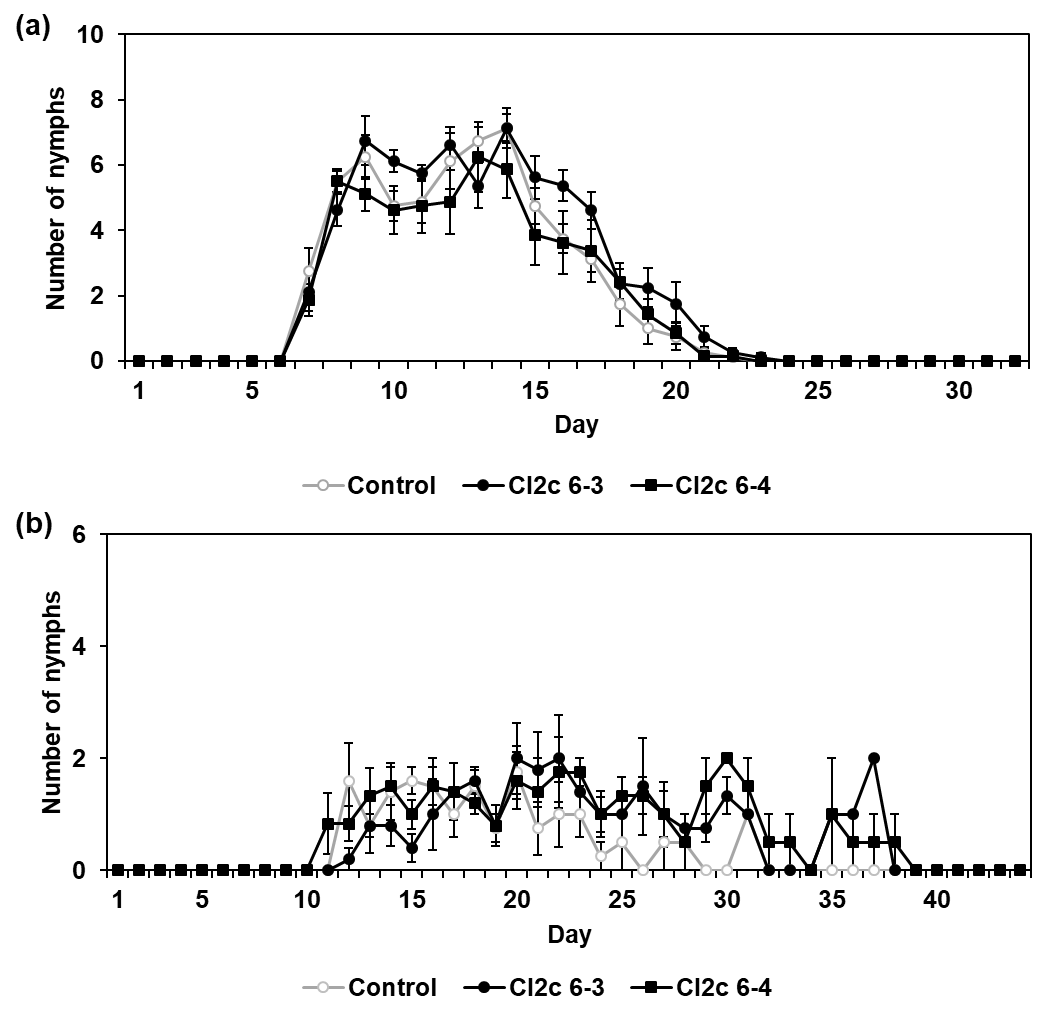


**S3 Fig. Nymph production per aphid during the lifespan of BCA and GPA**. (a) BCA, (b) GPA. All newborn nymphs were counted and removed each day of the reproductive period. Nymph production by GPA at late stages refers to a small number of long-lived aphids. Error bars indicate SE; n=8 for BCA, n=6 for GPA.
