# Supplementary material for: Overexpression of the aphid-induced serine protease inhibitor CI2c gene in barley affects the generalist green peach aphid, not the specialist bird cherry-oat aphid
Source: PLoS One. 2018 Mar 19;13(3):e0193816. doi: 10.1371/journal.pone.0193816 (PMC5858787; doi:10.1371/journal.pone.0193816)
Supplement: S4 Fig — (DOCX) [file pone.0193816.s005.docx]

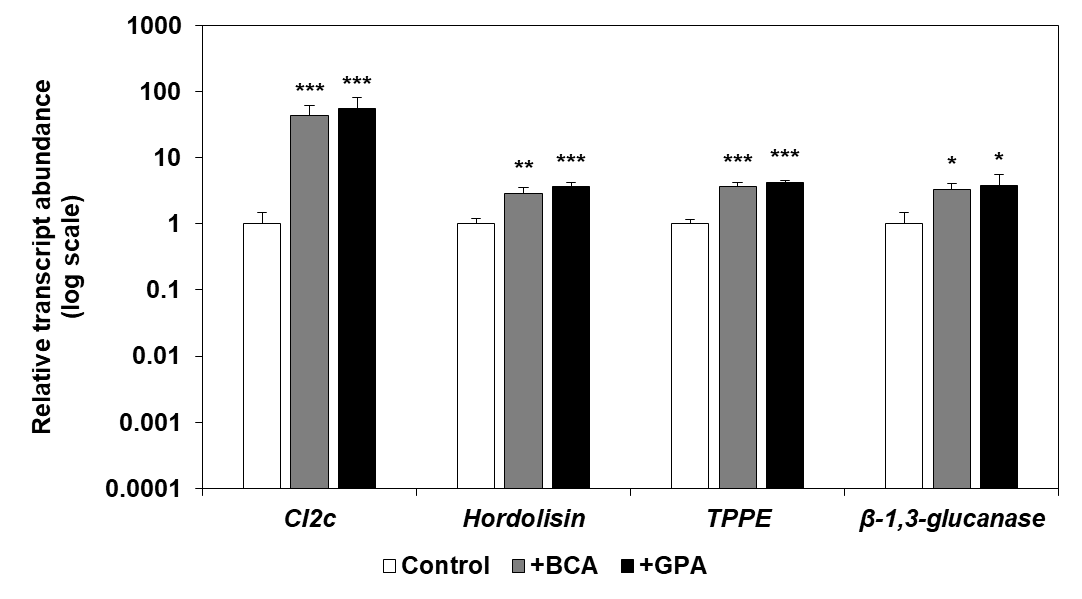


S4 Fig. Transcript abundance of aphid-induced genes in azygous control barley leaves before and after infestation with BCA and GPA. Primary leaves were infested during 48 h with twenty adult apterous BCA (grey bars) or GPA (black bars). White bars represent uninfested plants (±SE). The transcript abundance was calculated relative to two reference genes: *Hsp70* and *SF427* and for each gene normalized to uninfested plants set as 1.00. Asterisks indicate significant differences (Kruskal-Wallis test, **p*≤0.05, ***p*≤0.01, ****p*≤0.001). Six biological replicates (with two plants each) with three technical replicates.
